#  A Note on Likelihood Ratio Tests for Models with Latent Variables

**DOI:** 10.1007/s11336-020-09735-0

**Published:** 2020-12-21

**Authors:** Yunxiao Chen, Irini Moustaki, Haoran Zhang

**Affiliations:** 1 Department of Statistics, London School of Economics and Political Science, London, UK; 2 Shanghai Center for Mathematical Sciences, Fudan University, Shanghai, China

**Keywords:** Wilks’ theorem, $\chi ^2$ distribution, latent variable models, random effects models, dimensionality, tangent cone

## Abstract

The likelihood ratio test (LRT) is widely used for comparing the relative fit of nested latent variable models. Following Wilks’ theorem, the LRT is conducted by comparing the LRT statistic with its asymptotic distribution under the restricted model, a $\chi ^2$ distribution with degrees of freedom equal to the difference in the number of free parameters between the two nested models under comparison. For models with latent variables such as factor analysis, structural equation models and random effects models, however, it is often found that the $\chi ^2$ approximation does not hold. In this note, we show how the regularity conditions of Wilks’ theorem may be violated using three examples of models with latent variables. In addition, a more general theory for LRT is given that provides the correct asymptotic theory for these LRTs. This general theory was first established in Chernoff (J R Stat Soc Ser B (Methodol) 45:404–413, 1954) and discussed in both van der Vaart (Asymptotic statistics, Cambridge, Cambridge University Press, 2000) and Drton (Ann Stat 37:979–1012, 2009), but it does not seem to have received enough attention. We illustrate this general theory with the three examples.

**Electronic supplementary material:**

The online version of this article (10.1007/s11336-020-09735-0) contains supplementary material, which is available to authorized users.

## Introduction

### Literature on Likelihood Ratio Test

The likelihood ratio test (LRT) is one of the most popular methods for comparing nested models. When comparing two nested models that satisfy certain regularity conditions, the *p*-value of an LRT is obtained by comparing the LRT statistic with a $\chi ^2$ distribution with degrees of freedom equal to the difference in the number of free parameters between the two nested models. This reference distribution is suggested by the asymptotic theory of LRT that is known as Wilks’ theorem (Wilks 1938).

However, for the statistical inference of models with latent variables (e.g., factor analysis, item factor analysis for categorical data, structural equation models, random effects models, finite mixture models), it is often found that the $\chi ^2$ approximation suggested by Wilks’ theorem does not hold. There are various published studies showing that the LRT is not valid under certain violations/conditions (e.g., small sample size, wrong model under the alternative hypothesis, large number of items, non-normally distributed variables, unique variances equal to zero, lack of identifiability), leading to over-factoring and over-rejections; see, e.g., Hakstian et al. (1982), Liu and Shao (2003), Hayashi et al. (2007), Asparouhov and Muthén (2009), Wu and Estabrook (2016), Deng et al. (2018), Shi et al. (2018), Yang et al. (2018) and Auerswald and Moshagen (2019). There is also a significant amount of the literature on the effect of testing at the boundary of parameter space that arise when testing the significance of variance components in random effects models as well as in structural equation models (SEM) with linear or nonlinear constraints (see Stram and Lee 1994, 1995; Dominicus et al. 2006; Savalei and Kolenikov 2008; Davis-Stober 2009; Wu and Neale 2013; Du and Wang 2020).

Theoretical investigations have shown that certain regularity conditions of Wilks’ theorem are not always satisfied when comparing nested models with latent variables. Takane et al. (2003) and Hayashi et al. (2007) were among the ones who pointed out that models for which one needs to select dimensionality (e.g., principal component analysis, latent class, factor models) have points of irregularity in their parameter space that in some cases invalidate the use of LRT. Specifically, such issues arise in factor analysis when comparing models with different number of factors rather than comparing a factor model against the saturated model. The LRT for comparing a *q*-factor model against the saturated model does follow a $\chi ^2$ distribution under mild conditions. However, for nested models with different number of factors (*q*-factor model is the correct one against the one with $(q+k)$ factors), the LRT is likely not $\chi ^2$-distributed due to violation of one or more of the regularity conditions. This is in line with the two basic assumptions required by the asymptotic theory for factor analysis and SEM: the identifiability of the parameter vector and non-singularity of the information matrix (see Shapiro 1986 and references therein). More specifically, Hayashi et al. (2007) focus on exploratory factor analysis and on the problem that arises when the number of factors exceeds the true number of factors that might lead to rank deficiency and non-identifiability of model parameters. That corresponds to the violations of the two regularity conditions. Those findings go back to Geweke and Singleton (1980) and Amemiya and Anderson (1990). More specifically, Geweke and Singleton (1980) studied the behavior of the LRT in small samples and concluded that when the regularity conditions from Wilks’ theorem are not satisfied the asymptotic theory seems to be misleading in all sample sizes considered.

### Our Contributions

The contribution of this note is twofold. First, we provide a discussion about situations under which Wilks’ theorem for LRT may fail. Via three examples, we provide a relatively more complete picture about this issue in models with latent variables. Second, we introduce a unified asymptotic theory for LRT that covers Wilks’ theorem as a special case and provides the correct asymptotic reference distribution for LRT when Wilks’ theorem fails. This unified theory does not seem to have received enough attention in psychometrics, even though it has been established in statistics for long (Chernoff 1954; van der Vaart 2000; Drton 2009). In this note, we provide a tutorial on this theory, by presenting the theorems in a more accessible way and providing illustrative examples.

### Examples

To further illustrate the issue with the classical theory for LRT, we provide three examples. These examples suggest that the $\chi ^2$ approximation can perform poorly and give *p*-values that can be either more conservative or more liberal.

#### Example 1

**(Exploratory factor analysis)** Consider a dimensionality test in exploratory factor analysis (EFA). For ease of exposition, we consider two hypothesis testing problems: (a) testing a one-factor model against a two-factor model and (b) testing a one-factor model against a saturated multivariate normal model with an unrestricted covariance matrix. Similar examples have been considered in Hayashi et al. (2007) where similar phenomena have been studied.

**1(a).** Suppose that we have *J* mean-centered continuous indicators, ${\mathbf {X}}= (X_1, ..., X_J)^\top$, which follow a *J*-variate normal distribution $N({\mathbf {0}}, {\varvec{\Sigma }})$. The one-factor model parameterizes ${\varvec{\Sigma }}$ as

$$\begin{aligned} {\varvec{\Sigma }} = {\mathbf {a}}_1{\mathbf {a}}_1^\top + {\varvec{\Delta }}, \end{aligned}$$

where ${\mathbf {a}}_1 = (a_{11}, ..., a_{J1})^\top$ contains the loading parameters and ${\varvec{\Delta }} = diag(\delta _1, ..., \delta _J)$ is diagonal matrix with a diagonal entries $\delta _1$, ..., $\delta _J$. Here, ${\varvec{\Delta }}$ is the covariance matrix for the unique factors. Similarly, the two-factor model parameterizes ${\varvec{\Sigma }}$ as

$$\begin{aligned} {\varvec{\Sigma }} = {\mathbf {a}}_1{\mathbf {a}}_1^\top + {\mathbf {a}}_2{\mathbf {a}}_2^\top + {\varvec{\Delta }}, \end{aligned}$$

where ${\mathbf {a}}_2 = (a_{12}, ..., a_{J2})^\top$ contains the loading parameters for the second factor and we set $a_{12} = 0$ to ensure model identifiability. Obviously, the one-factor model is nested within the two-factor model. The comparison between these two models is equivalent to test

$$\begin{aligned} H_0: {\mathbf {a}}_2 = {\mathbf {0}} \text{ versus } H_a: {\mathbf {a}}_2 \ne {\mathbf {0}}. \end{aligned}$$

If Wilks’ theorem holds, then under $H_0$ the LRT statistic should asymptotically follow a $\chi ^2$ distribution with $J-1$ degrees of freedom.

**Table 1:** Values of the true parameters for the simulations in Example 1.

$a_{11}$	$a_{21}$	$a_{31}$	$a_{41}$	$a_{51}$	$a_{61}$
1.17	1.87	1.42	1.71	1.23	1.78

$\delta _{1}$	$\delta _{2}$	$\delta _{3}$	$\delta _{4}$	$\delta _{5}$	$\delta _{6}$
1.38	0.85	1.46	0.78	1.24	0.60

**Fig. 1:**
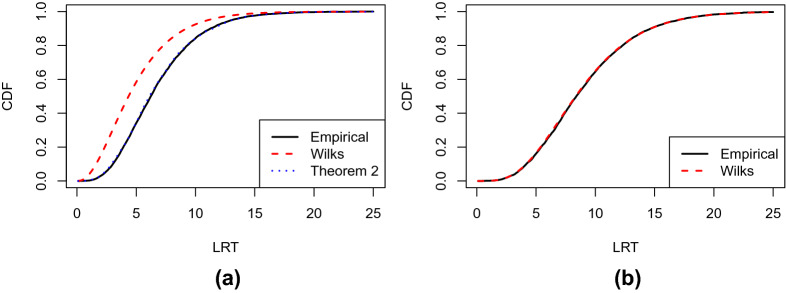
**a** Results of Example 1(a). The black solid line shows the empirical CDF of the LRT statistic, based on 5000 independent simulations. The red dashed line shows the CDF of the $\chi ^2$ distribution with 5 degrees of freedom as suggested by Wilks’ theorem. The blue dotted line shows the CDF of the reference distribution suggested by Theorem 2. **b** Results of Example 1(b). The black solid line shows the empirical CDF of the LRT statistic, and the red dashed line shows the CDF of the $\chi ^2$ distribution with 9 degrees of freedom as suggested by Wilks’ theorem

We now provide a simulated example. Data are generated from a one-factor model, with $J = 6$ indicators and $N=5000$ observations. The true parameter values are given in Table 1. We generate 5000 independent datasets. For each dataset, we compute the LRT for comparing the one- and two-factor models. Results are presented in panel (a) of Fig. 1. The black solid line shows the empirical cumulative distribution function (CDF) of the LRT statistic, and the red dashed line shows the CDF of the $\chi ^2$ distribution suggested by Wilks’ Theorem. A substantial discrepancy can be observed between the two CDFs. Specifically, the $\chi ^2$ CDF tends to stochastically dominate the empirical CDF, implying that p-values based on this $\chi ^2$ distribution tend to be more liberal. In fact, if we reject $H_0$ at 5% significance level based on these p-values, the actual type I error is 10.8%. These results suggest the failure of Wilks’ theorem in this example.

**1(b).** When testing the one-factor model against the saturated model, the LRT statistic is asymptotically $\chi ^2$ if Wilks’ theorem holds. The degrees of freedom of the $\chi ^2$ distribution are $J(J+1)/2 - 2J$, where $J(J+1)/2$ is the number of free parameters in an unrestricted covariance matrix ${\varvec{\Sigma }}$ and 2*J* is the number of parameters in the one-factor model. In panel (b) of Fig. 1, the black solid line shows the empirical CDF of the LRT statistic based on 5000 independent simulations, and the red dashed line shows the CDF of the $\chi ^2$ distribution with 9 degrees of freedom. As we can see, the two curves almost overlap with each other, suggesting that Wilks’ theorem holds here.

#### Example 2

**(Exploratory item factor analysis)** We further give an example of exploratory item factor analysis (IFA) for binary data, in which similar phenomena as those in Example 1 are observed. Again, we consider two hypothesis testing problems: (a) testing a one-factor model against a two-factor model and (b) testing a one-factor model against a saturated multinomial model for a binary random vector.

**2(a).** Suppose that we have a *J*-dimensional response vector, ${\mathbf {X}}= (X_1, ..., X_J)^\top$, where all the entries are binary-valued, i.e., $X_j \in \{0, 1\}$. It follows a categorical distribution, satisfying

$$\begin{aligned} P({\mathbf {X}}= {\mathbf {x}}) = \pi _{{\mathbf {x}}}, {\mathbf {x}}\in \{0,1\}^J, \end{aligned}$$

where $$\pi _{\mathbf {x}}\ge 0$$ and $$\sum _{{\mathbf {x}}\in \{0, 1\}^J} \pi _{{\mathbf {x}}} = 1$$.

The exploratory two-factor IFA model parameterizes $\pi _{\mathbf {x}}$ by

$$\begin{aligned} \pi _{{\mathbf {x}}} = \int \int \prod _{j=1}^J \frac{\exp (x_j(d_j + a_{j1}\xi _1 + a_{j2}\xi _2))}{1+\exp (d_j + a_{j1}\xi _1 + a_{j2}\xi _2)} \phi (\xi _1)\phi (\xi _2)d\xi _1d\xi _2, \end{aligned}$$

where $\phi (\cdot )$ is the probability density function of a standard normal distribution. This model is also known as a multidimensional two-parameter logistic (M2PL) model (Reckase 2009). Here, $a_{jk}$s are known as the discrimination parameters and $d_j$s are known as the easiness parameters. We denote ${\mathbf {a}}_1 = (a_{11},...,a_{J1})^\top$ and ${\mathbf {a}}_2 = (a_{12},...,a_{J2})^\top .$ For model identifiability, we set $a_{12} = 0$. When $a_{j2} = 0$, $j=2, ..., J$, then the two-factor model degenerates to the one-factor model. Similar to Example 1(a), if Wilks’ theorem holds, the LRT statistic should asymptotically follow a $\chi ^2$ distribution with $J-1$ degrees of freedom.

**Fig. 2:**
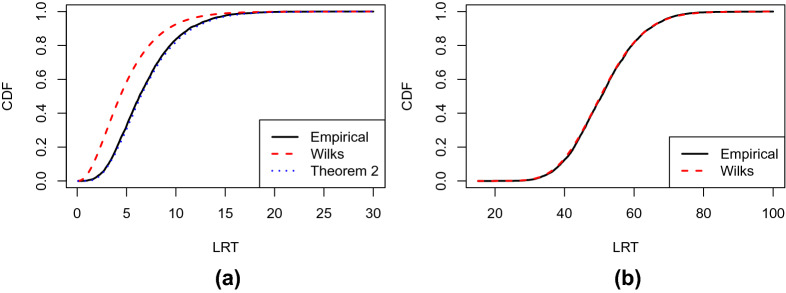
**a** Results of Example 2(a). The black solid line shows the empirical CDF of the LRT statistic, based on 5000 independent simulations. The red dashed line shows the CDF of the $\chi ^2$ distribution with 5 degrees of freedom as suggested by Wilks’ theorem. The blue dotted line shows the CDF of the reference distribution suggested by Theorem 2. **b** Results of Example 2(b). The black solid line shows the empirical CDF of the LRT statistic, and the red dashed line shows the CDF of the $\chi ^2$ distribution with 51 degrees of freedom as suggested by Wilks’ theorem

Simulation results suggest the failure of this $\chi ^2$ approximation. In Fig. 2, we provide plots similar to those in Fig. 1, based on 5000 datasets simulated from a one-factor IFA model with sample size $N = 5000$ and $J = 6$. The true parameters of this IFA model are given in Table 2. The result is shown in panel (a) of Fig. 2, where a similar pattern is observed as that in panel (a) of Fig. 1 for Example 1(a).

**Table 2:** Values of the true parameters for the simulations in Example 2.

$d_{1}$	$d_{2}$	$d_{3}$	$d_{4}$	$d_{5}$	$d_{6}$
$-$0.23	$-$0.12	0.07	0.31	$-$0.29	0.19

$a_{11}$	$a_{21}$	$a_{31}$	$a_{41}$	$a_{51}$	$a_{61}$
0.83	1.22	0.96	0.91	1.02	1.25

**2(b).** When testing the one-factor IFA model against the saturated model, the LRT statistic is asymptotically $\chi ^2$ if Wilks’ theorem holds, for which the degree of freedom is $2^J-1 - 2J$. Here, $2^J-1$ is the number of free parameters in the saturated model, and 2*J* is the number of parameters in the one-factor IFA model. The result is given in panel (b) of Fig. 2. Similar to Example 1(b), the empirical CDF and the CDF implied by Wilks’ theorem are very close to each other, suggesting that Wilks’ theorem holds here.

#### Example 3

**(Random effects model)** Our third example considers a random intercept model. Consider two-level data with individuals at level 1 nested within groups at level 2. Let $X_{ij}$ be data from the *j*th individual from the *i*th group, where $i = 1, ..., N$ and $j = 1, ..., J$. For simplicity, we assume all the groups have the same number of individuals. Assume the following random effects model,

$$\begin{aligned} X_{ij} = \beta _0 + \mu _i + \epsilon _{ij}, \end{aligned}$$

where $\beta _0$ is the overall mean across all the groups, $\mu _i \sim N(0, \sigma _1^2)$ characterizes the difference between the mean for group *i* and the overall mean, and $\epsilon _{ij} \sim N(0, \sigma _2^2)$ is the individual level residual.

To test for between-group variability under this model is equivalent to test

$$\begin{aligned} H_0: \sigma _1^2 = 0 \text{ against } H_a: \sigma _1^2 > 0. \end{aligned}$$

**Fig. 3:**
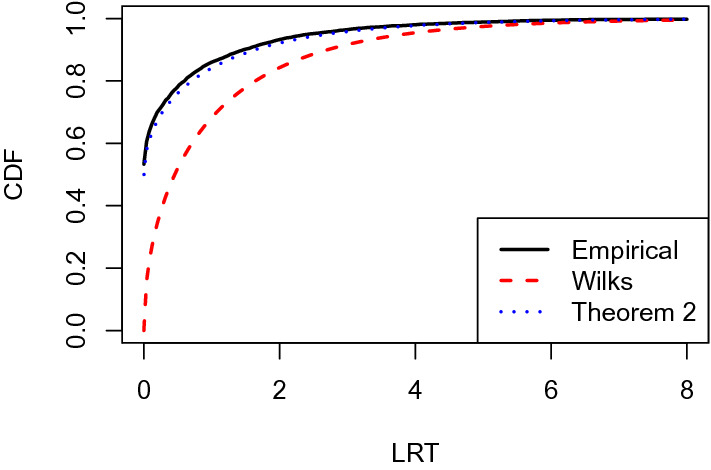
The black solid line shows the empirical CDF of the LRT statistic, based on 5000 independent simulations. The red dashed line shows the CDF of the $\chi ^2$ distribution with one degree of freedom as suggested by Wilks’ theorem. The blue dotted line shows the CDF of the mixture of $\chi ^2$ distribution suggested by Theorem 2 (Color figure online)

If Wilks’ theorem holds, then the LRT statistic should follow a $\chi ^2$ distribution with one degree of freedom. We conduct a simulation study and show the results in Fig. 3. In this figure, the black solid line shows the empirical CDF of the LRT statistic, based on 5000 independent simulations from the null model with $N = 200$, $J= 20$, $\beta _0 = 0$, and $\sigma ^2_2 = 1$. The red dashed line shows the CDF of the $\chi ^2$ distribution with one degree of freedom. As we can see, the two CDFs are not close to each other, and the empirical CDF tends to stochastically dominate the theoretical CDF suggested by Wilks’ theorem. It suggests the failure of Wilks’ theorem in this example.

This kind of phenomenon has been observed when the null model lies on the boundary of the parameter space, due to which the regularity conditions of Wilks’ theorem do not hold. The LRT statistic has been shown to often follow a mixture of $\chi ^2$ distribution asymptotically (e.g., Shapiro 1985; Self and Liang 1987), instead of a $\chi ^2$ distribution. As it will be shown in Sect. 2, such a mixture of $\chi ^2$ distribution can be derived from a general theory for LRT.

We now explain why Wilks’ theorem does not hold in Examples 1(a),  2(a), and  3. We define some generic notations. Suppose that we have i.i.d. observations ${\mathbf {X}}_1$, ..., ${\mathbf {X}}_N$, from a parametric model ${\mathcal {P}}_{\Theta } = \{P_{{\varvec{\theta }}}: {\varvec{\theta }} \in \Theta \subset {\mathbb {R}}^k\}$, where ${\mathbf {X}}_i = (X_{i1}, ..., X_{iJ})^\top .$ We assume that the distributions in $\mathcal P_{\Theta }$ are dominated by a common $\sigma$-finite measure $\nu$ with respect to which they have probability density functions $p_{{\varvec{\theta }}}: {\mathbb {R}}^J \rightarrow [0,\infty )$. Let $\Theta _0 \subset \Theta$ be a submodel and we are interested in testing

$$\begin{aligned} H_0: {\varvec{\theta }}\in \Theta _0 \text{ versus } H_a: {\varvec{\theta }}\in \Theta {\setminus } \Theta _0. \end{aligned}$$

Let $p_{{\varvec{\theta }}^* }$ be the true model for the observations, where ${\varvec{\theta }}^* \in \Theta _0$.

The likelihood function is defined as

$$\begin{aligned} l_N({\varvec{\theta }}) = \sum _{i=1}^N \log p_{{\varvec{\theta }}}({\mathbf {X}}_i), \end{aligned}$$

and the LRT statistic is defined as

$$\begin{aligned} \lambda _N = 2\left( \sup _{{\varvec{\theta }}\in \Theta } l_N({\varvec{\theta }}) - \sup _{{\varvec{\theta }}\in \Theta _0} l_N({\varvec{\theta }})\right) . \end{aligned}$$

Under suitable regularity conditions, Wilks’ theorem suggests that the LRT statistic $\lambda _N$ is asymptotically $\chi ^2$.

Wilks’ theorem for LRT requires several regularity conditions; see, e.g., Theorem 12.4.2, Lehmann and Romano (2006). Among these conditions, there are two conditions that the previous examples do not satisfy. First, it is required that ${\varvec{\theta }}^*$ is an interior point of $\Theta$. This condition is not satisfied for Example 3, when $\Theta$ is taken to be $\{(\beta _0, \sigma _1^2, \sigma _2^2): \beta _0 \in {\mathbb {R}}, \sigma _1^2 \in [0, \infty ), \sigma _2^2 \in [0, \infty )\}$, as the null model lies on the boundary of the parameter space. Second, it is required that the expected Fisher information matrix at ${\varvec{\theta }}^*$, $I({\varvec{\theta }}^*) = E_{{\varvec{\theta }}^*}[\nabla l_{N}({\varvec{\theta }}^*)\nabla l_{N}({\varvec{\theta }}^*)^\top ]/N$ is strictly positive definite. As we summarize in Lemma 1, this condition is not satisfied in Examples 1(a) and  2(a), when $\Theta$ is taken to be the parameter space of the corresponding two-factor model. However, interestingly, when comparing the one-factor model with the saturated model, the Fisher information matrix is strictly positive definite in Examples 1(b) and  2(b), for both simulated examples.

#### Lemma 1

1. For the two-factor model given in Example 1(a), choose the parameter space to be
  $$\begin{aligned} \Theta = \left\{ (\delta _1,...,\delta _J, a_{11},...,a_{J1},a_{22},...,a_{J2})^\top \in {\mathbb {R}}^{3J-1}:\delta _j >0, ~j=1,...,J \right\} . \end{aligned}$$
  If the true parameters satisfy $a^*_{j2}=0, ~j=2,...,J,$ then $I({\varvec{\theta }}^*)$ is non-invertible.
2. For the two-factor IFA model given in Example 2(a), choose the parameter space to be $\Theta = {\mathbb {R}}^{3J-1}.$ If the true parameters satisfy $a^*_{j2}=0, ~j=2,...,J,$ then $I({\varvec{\theta }}^*)$ is non-invertible.

We remark on the consequences of having a non-invertible information matrix. The first consequence is computational. If the information matrix is non-invertible, then the likelihood function does not tend to be strongly convex near the MLE, resulting in slow convergence. In the context of Examples 1(a) and  2(a), it means that computing the MLE for the corresponding two-factor models may have convergence issue. When convergence issue occurs, the obtained LRT statistic is below its actual value, due to the log likelihood for the two-factor model not achieving the maximum. Consequently, the *p*-value tends to be larger than its actual value, and thus, the decision based on the *p*-value tends to be more conservative than the one without convergence issue. This convergence issue is observed when conducting simulations for these examples. To improve the convergence, we use multiple random starting points when computing MLEs. The second consequence is a poor asymptotic convergence rate for the MLE. That is, the convergence rate is typically much slower than the standard parametric rate $N^{-1/2}$, even though the MLE is still consistent; see Rotnitzky et al. (2000) for more theoretical results on this topic.

We further provide some remarks on the LRT in Examples 1(b) and  2(b) that use a LRT for comparing the fitted model with the saturated model. Although Wilks’ theorem holds asymptotically in example 2(b), the $\chi ^2$ approximation may not always work well as in our simulated example. This is because, when the number of items becomes larger and the sample size is not large enough, the contingency table for all $2^J$ response patterns may be sparse, and thus, the saturated model cannot be accurately estimated. In that case, it is better to use a limited information inference method (e.g., Maydeu-Olivares and Joe 2005, 2006) as a goodness-of-fit test statistic. Similar issues might also occur to Example 1(b).

## General Theory for Likelihood Ratio Test

The previous discussions suggest that Wilks’ theorem does not hold for Examples 1(a),  2(a), and  3, due to the violation of regularity conditions. It is then natural to ask: What asymptotic distribution does $\lambda _N$ follow in these situations? Is there asymptotic theory characterizing such irregular situations? The answer to these questions is “yes.” In fact, a general theory characterizing these less regular situations has already been established in Chernoff (1954). In what follows, we provide a version of this general theory that is proved in van der Vaart (2000), Theorem 16.7. It is also given in Drton (2009), Theorem 2.6. Two problems will be considered, (1) comparing a submodel with the saturated model as in Examples 1(b) and  2(b), and (2) comparing two submodels as in Examples 1(a),  2(a), and  3.

### Testing Submodel Against Saturated Model

We first introduce a few notations. We use ${\mathbb {R}}^{J\times J}_{pd}$ and ${\mathbb {R}}^{J\times J}_d$ to denote the spaces of $J\times J$ strictly positive definite matrices and diagonal matrices, respectively. In addition, we define a one-to-one mapping $\rho$: $\mathcal {\mathbb {R}}_{pd}^{J\times J} \mapsto {\mathbb {R}}^{J(J+1)/2}$ that maps a positive definite matrix to a vector containing all its upper triangular entries (including the diagonal entries). That is, $\rho (\Sigma ) = (\sigma _{11}, \sigma _{12}..., \sigma _{1J}, \sigma _{22}, ..., \sigma _{2J}, ..., \sigma _{JJ})^\top$, for $\Sigma = (\sigma _{ij})_{J\times J} \in \mathcal {\mathbb {R}}_{pd}^{J\times J}$. We also define a one-to-one mapping $\mu$: ${\mathbb {R}}_{d}^{J\times J} \mapsto {\mathbb {R}}^{J}$ that maps a diagonal matrix to a vector containing all its diagonal entries.

We consider to compare a submodel versus the saturated model. Let $\Theta _0$ and $\Theta$ be the parameter spaces of the submodel and the saturated model, respectively, satisfying $\Theta _0 \subset \Theta \subset {\mathbb {R}}^k$. Also let ${\varvec{\theta }}^* \in \Theta _0$ be the true parameter vector. The asymptotic theory of the LRT for comparing $\Theta _0$ versus $\Theta$ requires regularity conditions C1-C5.

1. The true parameter vector ${\varvec{\theta }}^*$ is in the interior of $\Theta$.
2. There exists a measurable map $\dot{l}_{{\varvec{\theta }}}:{\mathbb {R}}^J \rightarrow {\mathbb {R}}^k$ such that
  1 $$\begin{aligned} \lim _{{\mathbf {h}}\rightarrow \mathbf{0}} \frac{1}{\Vert {\mathbf {h}}\Vert ^2} \int _{{\mathbb {R}}^J} \left( \sqrt{p_{{\varvec{\theta }}+{\mathbf {h}}}({\mathbf {x}})} - \sqrt{p_{{\varvec{\theta }}}({\mathbf {x}})} - \frac{1}{2}{\mathbf {h}}^\top \dot{l}_{{\varvec{\theta }}}({\mathbf {x}})\sqrt{p_{{\varvec{\theta }}}({\mathbf {x}})} \right) ^2 d\nu ({\mathbf {x}}) = 0, \end{aligned}$$
  and the Fisher information matrix $I({\varvec{\theta }}^*)$ for ${\mathcal {P}}_{\Theta }$ is invertible.
3. There exists a neighborhood of ${\varvec{\theta }}^*$, $U_{{\varvec{\theta }}^*} \subset \Theta$, and a measurable function $\dot{l}: {\mathbb {R}}^J \rightarrow {\mathbb {R}}$, square integrable as $\int _{{\mathbb {R}}^J}\dot{l}({\mathbf {x}})^2 dP_{{\varvec{\theta }}^*}({\mathbf {x}}) < \infty ,$ such that
  $$\begin{aligned} \vert \log p_{{\varvec{\theta }}_1}({\mathbf {x}}) - \log p_{{\varvec{\theta }}_2}({\mathbf {x}}) \vert \le \dot{l}({\mathbf {x}}) \Vert {\varvec{\theta }}_1 - {\varvec{\theta }}_2 \Vert , \quad \forall {\varvec{\theta }}_1,{\varvec{\theta }}_2 \in U_{{\varvec{\theta }}^*}. \end{aligned}$$
4. The maximum likelihood estimators (MLEs)
  $$\begin{aligned} {{\hat{{\varvec{\theta }}}}}_{N, \Theta } = \mathop {\mathrm{arg}\, \mathrm{max}}\limits _{{\varvec{\theta }}\in \Theta } l_N({\varvec{\theta }}) \end{aligned}$$
  and
  $$\begin{aligned} {{\hat{{\varvec{\theta }}}}}_{N, \Theta _0} = \mathop {\mathrm{arg}\, \mathrm{max}}\limits _{{\varvec{\theta }}\in \Theta _0} l_N({\varvec{\theta }}) \end{aligned}$$
  are consistent under $P_{{\varvec{\theta }}^*}.$

The asymptotic distribution of $\lambda _N$ depends on the local geometry of the parameter space $\Theta _0$ at ${\varvec{\theta }}^*$. This is characterized by the tangent cone $T_{\Theta _0}({\varvec{\theta }}^*)$, to be defined below.

#### Definition 1

The tangent cone $T_{\Theta _0}({\varvec{\theta }}^*)$ of the set $\Theta _0 \subset {\mathbb {R}}^k$ at the point ${\varvec{\theta }}^* \in {\mathbb {R}}^k$ is the set of vectors in ${\mathbb {R}}^k$ that are limits of sequences $\alpha _n({\varvec{\theta }}_n - {\varvec{\theta }}^*),$ where $\alpha _n$ are positive reals and ${\varvec{\theta }}_n \in \Theta _0$ converge to ${\varvec{\theta }}^*$.

The following regularity is required for the tangent cone $T_{\Theta _0}({\varvec{\theta }}^*)$ that is known as the Chernoff regularity.

C5. For every vector $\varvec{\tau }$ in the tangent cone $T_{\Theta _0}({\varvec{\theta }}^*)$, there exist $\epsilon >0$ and a map ${\varvec{\alpha }}:[0,\epsilon )\rightarrow \Theta _0$ with ${\varvec{\alpha }}(0) = {\varvec{\theta }}^*$ such that $\varvec{\tau }= \lim _{t\rightarrow 0+}[{\varvec{\alpha }}(t) - {\varvec{\alpha }}(0)]/t.$

Under the above regularity conditions, Theorem 1 holds and explains the phenomena in Examples 1(b) and  2(b).

#### Theorem 1

Suppose that conditions C1-C5 are satisfied for comparing nested models $\Theta _0 \subset \Theta \subset {\mathbb {R}}^k$, with ${\varvec{\theta }}^* \in \Theta _0$ being the true parameter vector. Then as *N* grows to infinity, the likelihood ratio statistic $\lambda _N$ converges to the distribution of

2 $$\begin{aligned} \min _{{\varvec{\tau }} \in T_{\Theta _0}({\varvec{\theta }}^*)} \Vert {\mathbf {Z}} - I({\varvec{\theta }}^*)^{\frac{1}{2}}{\varvec{\tau }} \Vert ^2, \end{aligned}$$

where ${\mathbf {Z}} = (Z_1, ..., Z_k)^\top$ is a random vector consisting of i.i.d. standard normal random variables.

#### Remark 1

We give some remarks on the regularity conditions. Conditions C1-C4 together ensure the asymptotic normality for $\sqrt{N}({\hat{{\varvec{\theta }}}}_{N,\Theta }-{\varvec{\theta }}^*)$. Condition C1 depends on both the true model and the saturated model. As will be shown below, this condition holds for the saturated models in Examples 1(b) and  2(b). Equation (1) in C2 is also known as the condition of “differentiable in quadratic mean” for ${\mathcal {P}}_{\Theta }$ at ${\varvec{\theta }}^*.$ If the map ${\varvec{\theta }}\mapsto \sqrt{p_{{\varvec{\theta }}}({\mathbf {x}})}$ is continuously differentiable for every ${\mathbf {x}},$ then C2 holds with $\dot{l}_{{\varvec{\theta }}}({\mathbf {x}}) = \frac{\partial }{\partial {\varvec{\theta }}}\log p_{{\varvec{\theta }}}({\mathbf {x}})$ (Lemma 7.6, van der Vaart (2000)). Furthermore, C3 holds if $\dot{l}({\mathbf {x}}) = \sup _{{\varvec{\theta }}\in U_{{\varvec{\theta }}^*}}\dot{l}_{{\varvec{\theta }}}({\mathbf {x}})$ is square integrable with respect to the measure $P_{{\varvec{\theta }}^*}.$ Specifically, if $\dot{l}({\mathbf {x}})$ is a bounded function, then C3 holds. C4 holds for our examples by Theorem 10.1.6, Casella and Berger (2002). C5 requires certain regularity on the local geometry of $T_{\Theta _0}({\varvec{\theta }}^*),$ which also holds for our examples below.

#### Remark 2

By Theorem 1, the asymptotic distribution for $\lambda _N$ depends on the tangent cone $T_{\Theta _0}({\varvec{\theta }}^*).$ If $T_{\Theta _0}({\varvec{\theta }}^*)$ is a linear subspace of ${\mathbb {R}}^k$ with dimension $k_0$, then one can easily show that the asymptotic reference distribution of $\lambda _N$ is $\chi ^2$ with degrees of freedom $k-k_0$. As we explain below, Theorem 1 directly applies to Examples 1(b) and 2(b). If $T_{\Theta _0}({\varvec{\theta }}^*)$ is a convex cone, then $\lambda _N$ converges to a mixture of $\chi ^2$ distribution (Shapiro 1985; Self and Liang 1987). That is, for any $x > 0$, $P(\lambda _N \le x)$ converges to $\sum _{i=0}^k w_k P(\xi _i \le x)$, as *N* goes to infinity, where $\xi _0 \equiv 0$ and $\xi _i$ follows a $\chi ^2$ distribution with *i* degrees of freedom for $i > 0$. Moreover, the weights sum up to 1/2 for the components with even degrees of freedom, and so do the weights for the components with odd degrees of freedom (Shapiro 1985).

#### Example 4

**(Exploratory factor analysis, revisited)** Now we consider Example 1(b). As the saturated model is a *J*-variate normal distribution with an unrestricted covariance matrix, its parameter space can be chosen as

$$\begin{aligned} \Theta = \{ \rho ({\varvec{\Sigma }}) : {\varvec{\Sigma }} \in {\mathbb {R}}_{pd}^{J\times J} \} \subset {\mathbb {R}}^{J(J+1)/2}, \end{aligned}$$

and the parameter space for the restricted model is

$$\begin{aligned} \Theta _0 = \left\{ \rho ({\varvec{\Sigma }}): {\varvec{\Sigma }} = {\mathbf {a}}_1{\mathbf {a}}_1^\top + {\varvec{\Delta }},~ {\mathbf {a}}_1 \in {\mathbb {R}}^J, {\varvec{\Delta }} \in {\mathbb {R}}_{pd}^{J\times J} \cap {\mathbb {R}}_{d}^{J\times J} \right\} . \end{aligned}$$

Suppose ${\varvec{\theta }}^* = \rho ({\varvec{\Sigma }}^*) \in \Theta _0,$ where ${\varvec{\Sigma }}^* = {{\mathbf {a}}_1^*}{{\mathbf {a}}_1^*}^\top + {\varvec{\Delta }}^*.$ It is easy to see that C1 holds with the current choice of $\Theta .$ The tangent cone $T_{\Theta _0}({\varvec{\theta }}^*)$ takes the form:

$$\begin{aligned} T_{\Theta _0}({\varvec{\theta }}^*) = \left\{ \rho ({\varvec{\Sigma }}): {\varvec{\Sigma }} = {{\mathbf {a}}_1^*}{{\mathbf {b}}}_1^\top + {{\mathbf {b}}}_1{{\mathbf {a}}_1^*}^\top + {\mathbf {B}}, ~ {{\mathbf {b}}}_1 \in {\mathbb {R}}^{J}, {\mathbf {B}} \in {\mathbb {R}}_{d}^{J\times J} \right\} , \end{aligned}$$

which is a linear subspace of ${\mathbb {R}}^{J(J+1)/2}$ with dimension 2*J*,  as long as $a^*_{j1} \ne 0, ~j=1,...,J.$ By Theorem 1, $\lambda _N$ converges to the $\chi ^2$ distribution with degrees of freedom $J(J+1)/2 - 2J.$

#### Example 5

**(Exploratory item factor analysis, revisited)** Now we consider Example 2(b). As the saturated model is a $2^J$-dimensional categorical distribution, its parameter space can be chosen as

$$\begin{aligned} \Theta = \left\{ {\varvec{\theta }}= \{\theta _{{\mathbf {x}}}\}_{{\mathbf {x}}\in \Gamma _J}: \theta _{{\mathbf {x}}} \ge 0, \sum _{{\mathbf {x}}\in \Gamma _J}\theta _{{\mathbf {x}}} \le 1 \right\} \subset {\mathbb {R}}^{2^J - 1}, \end{aligned}$$

where $\Gamma _J := \{0,1\}^J \backslash \{(0,...,0)^\top \}.$ Then, the parameter space for the restricted model is

3 $$\begin{aligned} \begin{aligned} \Theta _0 = \left\{ {\varvec{\theta }}\in \Theta : \theta _{{\mathbf {x}}} = \int \prod _{j=1}^J \frac{\exp (x_{j}(d_j + a_{j1}\xi _1))}{1+\exp (d_j + a_{j1}\xi _1)} \phi (\xi _1)d\xi _1, ~ {\mathbf {a}}_1,{{\mathbf {d}}}\in {\mathbb {R}}^J \right\} . \end{aligned} \end{aligned}$$

Let ${\varvec{\theta }}^* \in \Theta _0$ that corresponds to true item parameters ${\mathbf {a}}_1^* = (a^*_{j1},...,a^*_{J1})^\top$ and ${{\mathbf {d}}}^* = (d^*_{1},...,d^*_{J})^\top .$ By the form of $\Theta _0,$ ${\varvec{\theta }}^*$ is an interior point of $\Theta .$

For any ${\mathbf {x}}\in \Gamma _J,$ we define ${\mathbf {f}}_{\mathbf {x}}= (f_{1}({\mathbf {x}}),...,f_{J}({\mathbf {x}}))^\top$ and ${\mathbf {g}}_{{\mathbf {x}}} = (g_{1}({\mathbf {x}}),...,g_{J}({\mathbf {x}}))^\top ,$ where

$$\begin{aligned} f_{l}({\mathbf {x}}) = \int \prod _{j=1}^J \frac{\exp (x_{j}(d^*_{j} + a^*_{j1}\xi _1))}{1+\exp (d^*_{j} + a^*_{j1}\xi _1)} \left[ x_l - \frac{\exp (d^*_{l} + a^*_{l1}\xi _1)}{1+\exp (d^*_{l} + a^*_{l1}\xi _1)} \right] \phi (\xi _1) d\xi _1, \end{aligned}$$

and

$$\begin{aligned} g_{l}({\mathbf {x}}) = \int \prod _{j=1}^J \frac{\exp (x_{j}(d^*_{j} + a^*_{j1}\xi _1))}{1+\exp (d^*_{j} + a^*_{j1}\xi _1)} \left[ x_l - \frac{\exp (d^*_{l} + a^*_{l1}\xi _1)}{1+\exp (d^*_{l} + a^*_{l1}\xi _1)} \right] \xi _1\phi (\xi _1) d\xi _1, \end{aligned}$$

for $l=1,...,J.$ Then the tangent cone $T_{\Theta _0}({\varvec{\theta }}^*)$ has the form

$$\begin{aligned} T_{\Theta _0}({\varvec{\theta }}^*) = \left\{ {\varvec{\theta }}= \{\theta _{{\mathbf {x}}}\}_{{\mathbf {x}}\in \Gamma _J}: \theta _{{\mathbf {x}}} = {{\mathbf {b}}}_0^\top {\mathbf {f}}_{{\mathbf {x}}} + {{\mathbf {b}}}_1^\top {\mathbf {g}}_{{\mathbf {x}}},~{{\mathbf {b}}}_0,{{\mathbf {b}}}_1\in {\mathbb {R}}^J \right\} , \end{aligned}$$

which is a linear subspace of ${\mathbb {R}}^{2^J-1}$ with dimension 2*J*. By Theorem 1, $\lambda _N$ converges to the distribution of $\chi ^2$ with degrees of freedom $2^{J}-1 - 2J.$

### Comparing Two Nested Submodels

Theorem 1 is not applicable to Example 3, because ${\varvec{\theta }}^*$ is on the boundary of $\Theta$ if $\Theta$ is chosen to be $\{(\beta _0, \sigma _1^2, \sigma _2^2): \beta _0 \in {\mathbb {R}}, \sigma _1^2 \in [0, \infty ), \sigma _2^2 \in [0, \infty )\},$ and thus, C1 is violated. Theorem 1 is also not applicable to Examples 1(a) and  2(a), because the Fisher information matrix is not invertible when $\Theta$ is chosen to be the parameter space of the two-factor EFA and IFA models, respectively, in which case condition C2 is violated.

To derive the asymptotic theory for such problems, we view them as a problem of testing nested submodels under a saturated model for which ${\varvec{\theta }}^*$ is an interior point of $\Theta$ and the information matrix is invertible. Consider testing

$$\begin{aligned} H_0: {\varvec{\theta }}\in \Theta _0 \text{ versus } H_a: {\varvec{\theta }}\in \Theta _1{\setminus } \Theta _0, \end{aligned}$$

where $\Theta _0$ and $\Theta _1$ are two nested submodels of a saturated model $\Theta$, satisfying $\Theta _0 \subset \Theta _1 \subset \Theta \subset {\mathbb {R}}^{k}$. Under this formulation, Theorem 2 provides the asymptotic theory for the LRT statistic $\lambda _N = 2\left( \sup _{{\varvec{\theta }}\in \Theta _1} l_N({\varvec{\theta }}) - \sup _{{\varvec{\theta }}\in \Theta _0} l_N({\varvec{\theta }})\right)$.

To obtain the asymptotic distribution of $\lambda _N$, regularity conditions C1-C5 are still required for $\Theta _0 \subset \Theta$. Two additional conditions are needed for $\Theta _1$, which are satisfied for Examples 6, 7 and 8.

C6. The MLE under $\Theta _1$, ${{\hat{{\varvec{\theta }}}}}_{N, \Theta _1} = \mathop {\mathrm{arg}\, \mathrm{max}}\limits _{{\varvec{\theta }}\in \Theta _1} l_N({\varvec{\theta }})$, is consistent under $P_{{\varvec{\theta }}^*}.$
C7. Let $T_{\Theta _1}({\varvec{\theta }}^*)$ be the tangent cone for $\Theta _1$, defined the same as in Definition 1, but with $\Theta _0$ replaced by $\Theta _1$. $T_{\Theta _1}({\varvec{\theta }}^*)$ satisfies Chernoff regularity. That is, for every vector $\varvec{\tau }$ in the tangent cone $T_{\Theta _1}({\varvec{\theta }}^*)$ there exist $\epsilon >0$ and a map ${\varvec{\alpha }}:[0,\epsilon )\rightarrow \Theta _1$ with ${\varvec{\alpha }}(0) = {\varvec{\theta }}^*$ such that $\varvec{\tau }= \lim _{t\rightarrow 0+}[{\varvec{\alpha }}(t) - {\varvec{\alpha }}(0)]/t.$

#### Theorem 2

Let ${\varvec{\theta }}^* \in \Theta _0$ be the true parameter vector. Suppose that conditions C1-C7 are satisfied. As *N* grows to infinity, the likelihood ratio statistic $\lambda _N$ converges to the distribution of

4 $$\begin{aligned} \begin{aligned} \min _{{\varvec{\tau }} \in T_{\Theta _0}({\varvec{\theta }}^*)} \Vert {\mathbf {Z}} - I({\varvec{\theta }}^*)^{\frac{1}{2}}{\varvec{\tau }} \Vert ^2 - \min _{{\varvec{\tau }} \in T_{\Theta _1}({\varvec{\theta }}^*)} \Vert {\mathbf {Z}} - I({\varvec{\theta }}^*)^{\frac{1}{2}}{\varvec{\tau }} \Vert ^2, \end{aligned} \end{aligned}$$

where ${\mathbf {Z}} = (Z_1, ..., Z_k)^\top$ is a random vector consisting of i.i.d. standard normal random variables, and $I({\varvec{\theta }}^*)^{\frac{1}{2}}$ satisfies $I({\varvec{\theta }}^*)^{\frac{1}{2}} (I({\varvec{\theta }}^*)^{\frac{1}{2}})^\top = I({\varvec{\theta }}^*)$ that can be obtained by eigenvalue decomposition.

#### Example 6

**(Random effects model, revisited)** Now we consider Example 3. Let ${{\mathbf {1}}}_n$ denote a length-*n* vector whose entries are all 1 and ${{\mathbf {I}}}_n$ denote the $n\times n$ identity matrix. As ${\mathbf {X}}_i = (X_{i1},...,X_{iJ})^\top$ from the random effects model is multivariate normal with mean $\beta _0{{\mathbf {1}}}_J$ and covariance matrix $\sigma _1^2{{\mathbf {1}}}_J{{\mathbf {1}}}_J^\top + \sigma _2^2{{\mathbf {I}}}_J,$ the saturated parameter space can be taken as

$$\begin{aligned} \Theta = \{ (\rho ({\varvec{\Sigma }})^\top ,\beta _0)^\top : {\varvec{\Sigma }} \in {\mathbb {R}}^{J\times J}_{pd}, \beta _0\in {\mathbb {R}}\}. \end{aligned}$$

The parameter space for restricted models are

$$\begin{aligned} \Theta _0 = \{ (\rho ({\varvec{\Sigma }})^\top ,\beta _0)^\top :{\varvec{\Sigma }} = \sigma _2^2{{\mathbf {I}}}_J,~ \sigma _2^2 >0, \beta _0\in {\mathbb {R}}\}, \end{aligned}$$

and

$$\begin{aligned} \Theta _1 = \{ (\rho ({\varvec{\Sigma }})^\top ,\beta _0)^\top :{\varvec{\Sigma }} = \sigma _1^2{{\mathbf {1}}}_J{{\mathbf {1}}}_J^\top + \sigma _2^2{{\mathbf {I}}}_J, \sigma _1^2\ge 0, \sigma _2^2>0, \beta _0\in {\mathbb {R}}\}. \end{aligned}$$

Let ${\varvec{\theta }}^* = (\rho ({\varvec{\Sigma }}^*),\beta ^*_0) \in \Theta _0,$ where ${\varvec{\Sigma }}^* = {\sigma _2^*}^2{{\mathbf {I}}}_J.$ Then, C1 holds. The tangent cones for $\Theta _0$ and $\Theta _1$ are

$$\begin{aligned} T_{\Theta _0}({\varvec{\theta }}^*) = \{ (\rho ({\varvec{\Sigma }})^\top ,b_0)^\top :{\varvec{\Sigma }} = b_2{{\mathbf {I}}}_J,~ b_0,b_2\in {\mathbb {R}}\} \end{aligned}$$

and

$$\begin{aligned} T_{\Theta _1}({\varvec{\theta }}^*) = \{ (\rho ({\varvec{\Sigma }})^\top ,b_0)^\top :{\varvec{\Sigma }} = b_1{{\mathbf {1}}}_J{{\mathbf {1}}}_J^\top + b_2{{\mathbf {I}}}_J,~ b_1\ge 0, b_0,b_2\in {\mathbb {R}}\}. \end{aligned}$$

By Theorem 2, $\lambda _N$ converges to the distribution of (4).

In this example, the form of (4) can be simplified, thanks to the forms of $T_{\Theta _0}({\varvec{\theta }}^*)$ and $T_{\Theta _1}({\varvec{\theta }}^*).$ We denote

$$\begin{aligned} {\mathbf {c}}_0 = (0,...,0,1), ~{\mathbf {c}}_1 = (\rho ({{\mathbf {1}}}_J{{\mathbf {1}}}_J^\top )^\top ,0)^\top ,~{\mathbf {c}}_2 = (\rho ({{\mathbf {I}}}_J)^\top ,0)^\top \in {\mathbb {R}}^{J(J+1)/2+1}. \end{aligned}$$

It can be seen that $T_{\Theta _0}({\varvec{\theta }}^*)$ is a two-dimensional linear subspace spanned by $\{{\mathbf {c}}_0,{\mathbf {c}}_2\},$ and $T_{\Theta _1}({\varvec{\theta }}^*)$ is a half three-dimensional linear subspace defined as $\{\alpha _0{\mathbf {c}}_0+\alpha _1{\mathbf {c}}_1+\alpha _2{\mathbf {c}}_2: \alpha _1\ge 0,\alpha _0,\alpha _2\in {\mathbb {R}}\}.$ Let ${\mathbf {P}}_0$ denote the projection onto $T_{\Theta _0}({\varvec{\theta }}^*).$ Define

$$\begin{aligned} {\mathbf {v}}= \frac{{\mathbf {c}}_1 - {\mathbf {P}}_0{\mathbf {c}}_1}{\Vert {\mathbf {c}}_1 - {\mathbf {P}}_0{\mathbf {c}}_1\Vert }, \end{aligned}$$

and then, (4) has the form

5 $$\begin{aligned} \Vert {\mathbf {v}}^\top {\mathbf {Z}}\Vert ^21_{\{{\mathbf {v}}^\top {\mathbf {Z}}\ge 0\}}. \end{aligned}$$

It is easy to see that ${\mathbf {v}}^\top {\mathbf {Z}}$ follows standard normal distribution. Therefore, $\lambda _N$ converges to the distribution of $w^21_{\{w\ge 0\}},$ where *w* is a standard normal random variable. This is known as a mixture of $\chi ^2$ distribution. The blue dotted line in Fig. 3 shows the CDF of this mixture $\chi ^2$ distribution. This CDF is very close to the empirical CDF of the LRT, confirming our asymptotic theory.

#### Example 7

**(Exploratory factor analysis, revisited)** Now we consider Example 1(a). Let $\Theta ,\Theta _0,{\varvec{\theta }}^*$ and $T_{\Theta _0}({\varvec{\theta }}^*)$ be the same as those in Example 4. In addition, we define

$$\begin{aligned} \Theta _1 = \left\{ \rho ({\varvec{\Sigma }}): {\varvec{\Sigma }} = {\mathbf {a}}_1{\mathbf {a}}_1^\top + {\mathbf {a}}_2{\mathbf {a}}_2^\top + {\varvec{\Delta }},~ {\mathbf {a}}_1,{\mathbf {a}}_2 \in {\mathbb {R}}^J, a_{12}=0, {\varvec{\Delta }} \in {\mathbb {R}}_{pd}^{J\times J} \cap {\mathbb {R}}_{d}^{J\times J} \right\} . \end{aligned}$$

The tangent cone of $\Theta _1$ at ${\varvec{\theta }}^*$ becomes

$$\begin{aligned} T_{\Theta _1}({\varvec{\theta }}^*) = \left\{ \rho ({\varvec{\Sigma }}): {\varvec{\Sigma }} = {{\mathbf {a}}_1^*}{{\mathbf {b}}}_1^\top + {{\mathbf {b}}}_1{{\mathbf {a}}_1^*}^\top + {{\mathbf {b}}}_2{{\mathbf {b}}}_2^\top + {\mathbf {B}}, ~ {{\mathbf {b}}}_1,{{\mathbf {b}}}_2 \in {\mathbb {R}}^{J}, b_{12}=0, {\mathbf {B}} \in {\mathbb {R}}_{d}^{J\times J} \right\} . \end{aligned}$$

Note that $T_{\Theta _1}({\varvec{\theta }}^*)$ is not a linear subspace, due to the ${{\mathbf {b}}}_2{{\mathbf {b}}}_2^\top$ term. Therefore, by Theorem 2, the asymptotic distribution of $\lambda _N$ is not $\chi ^2$ . See the blue dotted line in Panel (a) of Fig. 1 for the CDF of this asymptotic distribution. This CDF almost overlaps with the empirical CDF of the LRT, suggesting that Theorem 2 holds here.

#### Example 8

**(Exploratory item factor analysis, revisited)** Now we consider Example 2(a). Let $\Theta ,\Theta _0,{\varvec{\theta }}^*$ and $T_{\Theta _0}({\varvec{\theta }}^*)$ be the same as those in Example 5. Let

$$\begin{aligned} \begin{aligned} \Theta _1 = \left\{ {\varvec{\theta }}\in \Theta : \theta _{{\mathbf {x}}} = \int \int \prod _{j=1}^J \frac{\exp (x_{j}(d_j + a_{j1}\xi _1+a_{j2}\xi _2))}{1+\exp (d_j + a_{j1}\xi _1+a_{j2}\xi _2)} \phi (\xi _1)\phi (\xi _2)d\xi _1d\xi _2, a_{12} = 0,{\mathbf {x}}\in \Gamma _J \right\} \end{aligned} \end{aligned}$$

be the parameter space for the two-factor model. Recall ${\mathbf {f}}_{{\mathbf {x}}}$ and ${\mathbf {g}}_{{\mathbf {x}}}$ as defined in Example 5. For any ${\mathbf {x}}\in \Gamma _J,$ we further define ${\mathbf {H}}_{{\mathbf {x}}} = (h_{rs}({\mathbf {x}}))_{J\times J},$ where

$$\begin{aligned} \begin{aligned} h_{rs}({\mathbf {x}}) =&\int \prod _{j=1}^J \frac{\exp (x_{j}(d^*_{j} + a^*_{j1}\xi _1))}{1+\exp (d^*_{j} + a^*_{j1}\xi _1)} \left[ x_r - \frac{\exp (d^*_{r} + a^*_{r1}\xi _1)}{1+\exp (d^*_{r} + a^*_{r1}\xi _1)} \right] \\&\times \left[ x_s - \frac{\exp (d^*_{s} + a^*_{s1}\xi _1)}{1+\exp (d^*_{s} + a^*_{s1}\xi _1)} \right] \phi (\xi _1)d\xi _1 \end{aligned} \end{aligned}$$

for $$r\ne s,$$ and

$$\begin{aligned} \begin{aligned} h_{rr}({\mathbf {x}}) =&\int \prod _{j=1}^J \frac{\exp (x_{j}(d^*_{j} + a^*_{j1}\xi _1))}{1+\exp (d^*_{j} + a^*_{j1}\xi _1)} \left\{ \left[ x_r - \frac{\exp (d^*_{r} + a^*_{r1}\xi _1)}{1+\exp (d^*_{r} + a^*_{r1}\xi _1)} \right] ^2 \right. \\&\left. - \frac{\exp (d_r^* + a_{r1}^*\xi _1)}{(1+\exp (d_r^* + a_{r1}^*\xi _1))^2} \right\} \phi (\xi _1)d\xi _1. \end{aligned} \end{aligned}$$

Then, the tangent cone of $\Theta _1$ at ${\varvec{\theta }}^*$ is

6 $$\begin{aligned} T_{\Theta _1}({\varvec{\theta }}^*) = \left\{ {\varvec{\theta }}=\{\theta _{{\mathbf {x}}}\}_{{\mathbf {x}}\in \Gamma _J}: \theta _{{\mathbf {x}}} = {{\mathbf {b}}}_0^\top {\mathbf {f}}_{{\mathbf {x}}} + {{\mathbf {b}}}_1^\top {\mathbf {g}}_{{\mathbf {x}}} + {{\mathbf {b}}}_2^\top {\mathbf {H}}_{{\mathbf {x}}}{{\mathbf {b}}}_2,~ {{\mathbf {b}}}_0,{{\mathbf {b}}}_1,{{\mathbf {b}}}_2 \in {\mathbb {R}}^J, b_{12} = 0 \right\} . \end{aligned}$$

Similar to Example 7, $T_{\Theta _1}({\varvec{\theta }}^*)$ is not a linear subspace, and thus, $\lambda _N$ is not asymptotically $\chi ^2$ . In Panel (a) of Fig. 2, the asymptotic CDF suggested by Theorem 2 is shown as the blue dotted line. Similar to the previously examples, this CDF is very close to the empirical CDF of the LRT.

## Discussion

In this note, we point out how the regularity conditions of Wilks’ theorem may be violated, using three examples of models with latent variables. In these cases, the asymptotic distribution of the LRT statistic is no longer $\chi ^2$, and therefore, the test may no longer be valid. It seems that the regularity conditions of Wilks’ theorem, especially the requirement on a non-singular Fisher information matrix, have not received enough attention. As a result, the LRT is often misused. Although we focus on LRT, it is worth pointing out that other testing procedures, including the Wald and score tests, as well as limited information tests (e.g., tests based on bivariate information), require similar regularity conditions and thus may also be affected.

We present a general theory for LRT first established in Chernoff (1954) that is not widely known in psychometrics and related fields. As we illustrate by the three examples, this theory applies to irregular cases not covered by Wilks’ theorem. There are other examples for which this general theory is useful. For example, Examples 1(a) and  2(a) can be easily generalized to the comparison of factor models with different numbers of factors, under both confirmatory and exploratory settings. This theory can also be applied to model comparison in latent class analysis that also suffers from a non-invertible information matrix. To apply the theorem, the key is to choose a suitable parameter space and then characterize the tangent cone at the true model.

There are alternative inference methods for making statistical inference under such irregular situations. One method is to obtain a reference distribution for LRT via parametric bootstrap. Under the same regularity conditions as in Theorem 2, we believe that the parametric bootstrap is still consistent. The parametric bootstrap may even achieve better approximation accuracy for finite sample data than the asymptotic distributions given by Theorems 1 and 2. However, for complex latent variable models (e.g., IFA models with many factors), the parametric bootstrap may be computationally intensive, due to the high computational cost of repeatedly computing the marginal maximum likelihood estimators. On the other hand, Monte Carlo simulation of the asymptotic distribution in Theorem 2 is computationally much easier, even though there are still optimizations to be solved. Another method is the split likelihood ratio test recently proposed by Wasserman et al. (2020) that is computationally fast and does not suffer from singularity or boundary issues. By making use of a sample splitting trick, this split LRT is able to control the type I error at any pre-specified level. However, it may be quite conservative sometimes.

This paper focuses on the situations where the true model is exactly a singular or boundary point of the parameter space. However, the LRT can also be problematic when the true model is near a singular or boundary point. A recent article by Mitchell et al. (2019) provides a treatment of this problem, where a finite sample approximating distribution is derived for LRT.

Besides the singularity and boundary issues, the asymptotic distribution may be inaccurate when the dimension of the parameter space is relatively high comparing with the sample size. This problem has been intensively studied in statistics and a famous result is the Bartlett correction which provides a way to improve the $\chi ^2$ approximation (Bartlett 1937; Bickel and Ghosh 1990; Cordeiro 1983; Box 1949; Lawley 1956; Wald 1943). When the regularity conditions do not hold, the classical form of Bartlett correction may no longer be suitable. A general form of Bartlett correction remains to be developed, which is left for future investigation.

### Electronic supplementary material

Below is the link to the electronic supplementary material.

Supplementary material 1 (pdf 147 KB)

## Appendix

### Proof of Lemma 1

Denote the (*i*, *j*)-entry of the Fisher information matrix $I({\varvec{\theta }}^*)$ as $q_{ij}.$ In both cases, we show that $q_{ij} = 0$ for $i \ge 2J+1,$ or $j \ge 2J+1,$ and therefore, $I({{\varvec{\theta }}^*})$ is non-invertible. Since

$$\begin{aligned} q_{ij} = \int \frac{\partial \log p_{{\varvec{\theta }}}\left( {\mathbf {x}}\right) }{\partial \theta _i} \Bigr |_{{\varvec{\theta }}^*}\frac{\partial \log p_{{\varvec{\theta }}}\left( {\mathbf {x}}\right) }{\partial \theta _j}\Bigr |_{{\varvec{\theta }}^*} p_{{\varvec{\theta }}^*}({\mathbf {x}})d{\mathbf {x}}, \end{aligned}$$

it suffices to show that

$$\begin{aligned} \frac{\partial \log p_{{\varvec{\theta }}}\left( {\mathbf {x}}\right) }{\partial \theta _i}\Bigr |_{{\varvec{\theta }}^*} = 0, \quad j \ge 2J+1. \end{aligned}$$

In the case of two-factor model, it suffices to show that

$$\begin{aligned} \frac{\partial \log p_{{\varvec{\theta }}}\left( {\mathbf {x}}\right) }{\partial a_{l2}} \Bigr |_{{\varvec{\theta }}^*} = 0, \end{aligned}$$

for $l=2,...,J.$ Let $\sigma _{ij}$ be the (*i*, *j*)-entry of the covariance matrix $\Sigma$ and it is easy to see that $\sigma _{ij} = a_{i1}a_{j1}+a_{i2}a_{j2}+1_{\{i=j\}}\delta _i,$ where $a_{12} = 0.$ By the chain rule,

$$\begin{aligned} \frac{\partial \log p_{{\varvec{\theta }}}\left( {\mathbf {x}}\right) }{\partial a_{l2}} = \sum _{i\le j} \frac{\partial \log p_{{\varvec{\theta }}}\left( {\mathbf {x}}\right) }{\partial \sigma _{ij}} \frac{\partial \sigma _{ij}}{\partial a_{l2}}. \end{aligned}$$

Since

$$\begin{aligned} \begin{aligned} \frac{\partial \sigma _{ij}}{\partial a_{l2}} \Bigr |_{{\varvec{\theta }}^*}&= 1_{\{l=i\}}a^*_{j2} + 1_{\{l=j\}}a^*_{i2}\\&= 0, \end{aligned} \end{aligned}$$

then $I({\varvec{\theta }}^*)$ is non-invertible in the case of two-factor model.

In the case of two-factor IFA model, since

$$\begin{aligned} \frac{\partial \log p_{{\varvec{\theta }}}\left( {\mathbf {x}}\right) }{\partial \theta _i} = \frac{1}{p_{{\varvec{\theta }}}({\mathbf {x}})}\frac{\partial p_{{\varvec{\theta }}}\left( {\mathbf {x}}\right) }{\partial a_{l2}} \end{aligned}$$

it suffices to show that

$$\begin{aligned} \frac{\partial p_{{\varvec{\theta }}}\left( {\mathbf {x}}\right) }{\partial a_{l2}} \Bigr |_{{\varvec{\theta }}^*} = 0, \end{aligned}$$

for $$l=2,...,J.$$ Since

$$\begin{aligned} \begin{aligned} \frac{\partial p_{{\varvec{\theta }}}\left( {\mathbf {x}}\right) }{\partial a_{l2}} \Bigr |_{{\varvec{\theta }}^*}&= \int \int \prod _{j=1}^J \frac{\exp (x_{j}(d^*_{j} + a^*_{j1}\xi _1))}{1+\exp (d^*_{j} + a^*_{j1}\xi _1)} \left[ x_l - \frac{\exp (d^*_{l} + a^*_{l1}\xi _1)}{1+\exp (d^*_{l} + a^*_{l1}\xi _1)} \right] \xi _2\phi (\xi _1)\phi (\xi _2) d\xi _1d\xi _2\\&= \int \xi _2 \phi (\xi _2) d\xi _2 \times \int \prod _{j=1}^J \frac{\exp (x_{j}(d^*_{j} + a^*_{j1}\xi _1))}{1+\exp (d^*_{j} + a^*_{j1}\xi _1)} \left[ x_l - \frac{\exp (d^*_{l} + a^*_{l1}\xi _1)}{1+\exp (d^*_{l} + a^*_{l1}\xi _1)} \right] \phi (\xi _1) d\xi _1 \\&=0, \end{aligned} \end{aligned}$$

then $I({\varvec{\theta }}^*)$ is non-invertible in the case of two-factor IFA model. $\square$
