# Supplementary material for: A Note on Likelihood Ratio Tests for Models with Latent Variables
Source: Psychometrika. 2020 Dec 21;85(4):996–1012. doi: 10.1007/s11336-020-09735-0 (PMC7826319; doi:10.1007/s11336-020-09735-0)
Supplement: Supplementary file 1 — Supplementary material 1 (pdf 147 KB) [file 11336_2020_9735_MOESM1_ESM.pdf]

Supplement to "A Note on Likelihood Ratio Tests for Models with Latent Variables"

Proof of Theorem 1. We refer readers to Drton (2009), Theorem 2.6. ■

Proof of Theorem 2. The proof is similar to that of Theorem 16.7, van der Vaart (2000). We only state the main steps and skip the details which readers can find in van der Vaart (2000).

We introduce some notations. Let

$$T_{N,0} = \left\{ \sqrt{N}(\boldsymbol{\theta} - \boldsymbol{\theta}^*) : \boldsymbol{\theta} \in \Theta_0 \right\}$$

and

$$T_{N,1} = \left\{ \sqrt{N}(\boldsymbol{\theta} - \boldsymbol{\theta}^*) : \boldsymbol{\theta} \in \Theta_1 \right\}.$$

Under conditions C5 and C7, $T_{N,0}, T_{N,1}$ converge to $T_{\Theta_0}(\boldsymbol{\theta}^*)$ and $T_{\Theta_1}(\boldsymbol{\theta}^*)$, respectively in the sense of van der Vaart (2000). Let $I(\boldsymbol{\theta}^*)^{-\frac{1}{2}}$ denote the inverse of $I(\boldsymbol{\theta}^*)^{\frac{1}{2}}$. Let

$\mathbb{G}_N = \sqrt{N}(\mathbb{P}_N - P_{\boldsymbol{\theta}^*})$ be the empirical process. Then,

$$\begin{aligned}
\lambda_N &= 2 \sup_{\boldsymbol{\theta} \in \Theta_1} l_N(\boldsymbol{\theta}) - 2 \sup_{\boldsymbol{\theta} \in \Theta_0} l_N(\boldsymbol{\theta}) \\
&= 2 \sup_{\mathbf{h} \in T_{N,1}} N\mathbb{P}_N \log p_{\boldsymbol{\theta}^* + \mathbf{h}/\sqrt{N}}(\mathbf{x}) - 2 \sup_{\mathbf{h} \in T_{N,0}} N\mathbb{P}_N \log p_{\boldsymbol{\theta}^* + \mathbf{h}/\sqrt{N}}(\mathbf{x}) \\
&= 2 \sup_{\mathbf{h} \in T_{N,1}} N\mathbb{P}_N \log \frac{p_{\boldsymbol{\theta}^* + \mathbf{h}/\sqrt{N}}(\mathbf{x})}{p_{\boldsymbol{\theta}^*}(\mathbf{x})} - 2 \sup_{\mathbf{h} \in T_{N,0}} N\mathbb{P}_N \log \frac{p_{\boldsymbol{\theta}^* + \mathbf{h}/\sqrt{N}}(\mathbf{x})}{p_{\boldsymbol{\theta}^*}(\mathbf{x})} \\
&= 2 \sup_{\mathbf{h} \in T_{N,1}} \left(\mathbf{h}^\top \mathbb{G}_N \dot{l}_{\boldsymbol{\theta}^*} - \frac{1}{2} \mathbf{h}^\top I(\boldsymbol{\theta}^*) \mathbf{h} \right) - 2 \sup_{\mathbf{h} \in T_{N,0}} \left(\mathbf{h}^\top \mathbb{G}_N \dot{l}_{\boldsymbol{\theta}^*} - \frac{1}{2} \mathbf{h}^\top I(\boldsymbol{\theta}^*) \mathbf{h} \right) + o_p(1) \quad (.1) \\
&= \sup_{\mathbf{h} \in T_{\Theta_0}(\boldsymbol{\theta}^*)} \left\| I(\boldsymbol{\theta}^*)^{-\frac{1}{2}} \mathbb{G}_N \dot{l}_{\boldsymbol{\theta}^*} - I(\boldsymbol{\theta}^*)^{\frac{1}{2}} \mathbf{h} \right\|^2 - \sup_{\mathbf{h} \in T_{\Theta_1}(\boldsymbol{\theta}^*)} \left\| I(\boldsymbol{\theta}^*)^{-\frac{1}{2}} \mathbb{G}_N \dot{l}_{\boldsymbol{\theta}^*} - I(\boldsymbol{\theta}^*)^{\frac{1}{2}} \mathbf{h} \right\|^2 + o_p(1). \quad (.2)
\end{aligned}$$

The $\dot{l}_{\boldsymbol{\theta}^*}$ is defined by condition C2. For details of (.1), see the proof of Theorem 16.7, van der Vaart (2000). (.2) is derived from

$$2\mathbf{h}^\top \mathbb{G}_N \dot{l}_{\boldsymbol{\theta}^*} - \mathbf{h}^\top I(\boldsymbol{\theta}^*) \mathbf{h} = -\left\| I(\boldsymbol{\theta}^*)^{-\frac{1}{2}} \mathbb{G}_N \dot{l}_{\boldsymbol{\theta}^*} - I(\boldsymbol{\theta}^*)^{\frac{1}{2}} \mathbf{h} \right\|^2 + \left\| I(\boldsymbol{\theta}^*)^{-\frac{1}{2}} \mathbb{G}_N \dot{l}_{\boldsymbol{\theta}^*} \right\|^2,$$

and the fact that $T_{N,0}, T_{N,1}$ converge to $T_{\Theta_0}(\boldsymbol{\theta}^*)$ and $T_{\Theta_1}(\boldsymbol{\theta}^*)$, respectively. By central limit theorem, $I(\boldsymbol{\theta}^*)^{-\frac{1}{2}} \mathbb{G}_N \dot{l}_{\boldsymbol{\theta}^*}$ converges to k-variate standard normal distribution. We complete the proof by continuous mapping theorem. ■

References

Drton, M. (2009). Likelihood ratio tests and singularities. *The Annals of Statistics*, 37:979–1012.

van der Vaart, A. W. (2000). *Asymptotic statistics*. Cambridge, England: Cambridge University Press.
